# Virtual Reality for Health Professions Education: Systematic Review and Meta-Analysis by the Digital Health Education Collaboration

**DOI:** 10.2196/12959

**Published:** 2019-01-22

**Authors:** Bhone Myint Kyaw, Nakul Saxena, Pawel Posadzki, Jitka Vseteckova, Charoula Konstantia Nikolaou, Pradeep Paul George, Ushashree Divakar, Italo Masiello, Andrzej A Kononowicz, Nabil Zary, Lorainne Tudor Car

**Affiliations:** 1 Family Medicine and Primary Care Lee Kong Chian School of Medicine Nanyang Technological University Singapore Singapore Singapore; 2 Health Services and Outcomes Research National Healthcare Group Singapore Singapore Singapore; 3 Centre for Population Health Sciences Lee Kong Chian School of Medicine Nanyang Technological University Singapore Singapore Singapore; 4 Faculty of Wellbeing, Education and Language Studies The Open University Milton Keynes United Kingdom; 5 Centre de Philosophie du Proit Universite Catholique de Louvain Louvain-la-Neuve Belgium; 6 Department of Clinical Science and Education Karolinska Institutet Stockholm Sweden; 7 Faculty of Social Sciences Linnaeus University Växjö Sweden; 8 Department of Bioinformatics and Telemedicine Jagiellonian University Medical College Krakow Poland; 9 Games for Health Innovations Centre Lee Kong Chian School of Medicine Nanyang Technological University Singapore Singapore Singapore; 10 Department of Learning, Informatics, Management and Ethics Karolinska Institutet Stockholm Sweden; 11 International Medical Simulation Centre, Mohammed VI University of Health Sciences Casablanca Morocco; 12 Department of Primary Care and Public Health School of Public Health Imperial College London London United Kingdom

**Keywords:** virtual reality, health professions education, randomized controlled trials, systematic review, meta-analysis

## Abstract

**Background:**

Virtual reality (VR) is a technology that allows the user to explore and manipulate computer-generated real or artificial three-dimensional multimedia sensory environments in real time to gain practical knowledge that can be used in clinical practice.

**Objective:**

The aim of this systematic review was to evaluate the effectiveness of VR for educating health professionals and improving their knowledge, cognitive skills, attitudes, and satisfaction.

**Methods:**

We performed a systematic review of the effectiveness of VR in pre- and postregistration health professions education following the gold standard Cochrane methodology. We searched 7 databases from the year 1990 to August 2017. No language restrictions were applied. We included randomized controlled trials and cluster-randomized trials. We independently selected studies, extracted data, and assessed risk of bias, and then, we compared the information in pairs. We contacted authors of the studies for additional information if necessary. All pooled analyses were based on random-effects models. We used the Grading of Recommendations, Assessment, Development and Evaluations (GRADE) approach to rate the quality of the body of evidence.

**Results:**

A total of 31 studies (2407 participants) were included. Meta-analysis of 8 studies found that VR slightly improves postintervention knowledge scores when compared with traditional learning (standardized mean difference [SMD]=0.44; 95% CI 0.18-0.69; I^2^=49%; 603 participants; moderate certainty evidence) or other types of digital education such as online or offline digital education (SMD=0.43; 95% CI 0.07-0.79; I^2^=78%; 608 participants [8 studies]; low certainty evidence). Another meta-analysis of 4 studies found that VR improves health professionals’ cognitive skills when compared with traditional learning (SMD=1.12; 95% CI 0.81-1.43; I^2^=0%; 235 participants; large effect size; moderate certainty evidence). Two studies compared the effect of VR with other forms of digital education on skills, favoring the VR group (SMD=0.5; 95% CI 0.32-0.69; I^2^=0%; 467 participants; moderate effect size; low certainty evidence). The findings for attitudes and satisfaction were mixed and inconclusive. None of the studies reported any patient-related outcomes, behavior change, as well as unintended or adverse effects of VR. Overall, the certainty of evidence according to the GRADE criteria ranged from low to moderate. We downgraded our certainty of evidence primarily because of the risk of bias and/or inconsistency.

**Conclusions:**

We found evidence suggesting that VR improves postintervention knowledge and skills outcomes of health professionals when compared with traditional education or other types of digital education such as online or offline digital education. The findings on other outcomes are limited. Future research should evaluate the effectiveness of immersive and interactive forms of VR and evaluate other outcomes such as attitude, satisfaction, cost-effectiveness, and clinical practice or behavior change.

## Introduction

Adequately trained health professionals are essential to ensure access to health services and to achieve universal health coverage [1]. In 2013, the World Health Organization (WHO) estimated a shortage of approximately 17.4 million health professionals worldwide [1]. The shortage and disproportionate distribution of health workers worldwide can be aggravated by the inadequacy of training programs (in terms of content, organization, and delivery) and experience needed to provide uniform health care services to all [2]. It has, therefore, become essential to generate strategies focused on scalable, efficient, and high-quality health professions education [3]. Increasingly, digital technology, with its pervasive use and relentless advancement, is seen as a promising source of effective and efficient health professions education and training systems [4].

Digital education (also known as eLearning) is the act of teaching and learning by means of digital technologies. It is an overarching term for an evolving multitude of educational approaches, concepts, methods, and technologies [5]. Digital education can include, but is not limited to, online and offline computer-based digital education, massive open online courses, virtual reality (VR), virtual patients, mobile learning, serious gaming and gamification, and psychomotor skills trainers [5]. A strong evidence base is needed to support effective use of these different digital modalities for health professions education. To this end, as part of an evidence synthesis series for digital health education, we focused on one of the digital education modalities, VR [6].

VR is a technology that allows the user to explore and manipulate computer-generated real or artificial three-dimensional (3D) multimedia sensory environments in real time. It allows for a first-person active learning experience through different levels of immersion; that is, a perception of the digital world as real and the ability to interact with objects and/or perform a series of actions in this digital world [7-9]. VR can be displayed with a variety of tools, including computer or mobile device screens, and VR rooms of head-mounted displays. VR rooms are projector-based immersive 3D visualization systems simulating real or virtual environments in a closed space and involve multiple users at the same time [10]. Head-mounted displays are placed over the user’s head and provide an immersive 3D environmental experience for learning [7]. VR can also facilitate diverse forms of health professions education. For example, it is often used for designing 3D anatomical structure models, which can be toggled and zoomed into [11]. VR also enables the creation of virtual worlds or 3D environments with virtual representations of users, called avatars. Avatars in VR for health professions education can represent patients or health professionals. By enabling simulation, VR is highly conducive to clinical and surgical procedures-focused training.

We found several reviews focusing primarily on the development of technical skills as part of surgical and clinical procedures-focused training, mostly calling for more research on the topic [12-15]. However, VR also offers a range of other educational opportunities, such as development of cognitive, nontechnical competencies [13-18]. Our review addresses this gap in the existing literature by investigating the effectiveness of VR for health professions education.

## Methods

### Systematic Review

We adhered to the published protocol [6] and followed the Cochrane guidelines [19]. The review is reported according to the Preferred Reporting Items for Systematic Reviews and Meta-Analyses guidelines [20]. For a detailed description of the methodology, please refer to the study by Car et al [5].

### Study Selection

We included randomized and cluster-randomized controlled trials that compared any VR intervention with any control intervention, for the education of pre- or postregistration health professionals. We included health professionals with qualifications found in the Health Field of Education and Training (091) of the International Standard Classification of Education. VR interventions could be delivered as the only mode of education intervention or blended with traditional learning (ie, blended learning). We included studies on VR for cognitive and nontechnical health professions education, including all VR delivery devices and levels of immersion. We included studies that reported VR as an intervention for healthcare professionals without the participant using any additional physical objects or devices such as probes or handles for psychomotor or technical skill development. We included studies that compared VR or blended learning with traditional learning, other types of digital educations, or another form of VR intervention.

We differentiated the following types of VR: 3D models, virtual patient or virtual health professional (VP or VHP) within VR and surgical simulation. Although we included studies including virtual patients in a VR, studies of virtual patient scenarios outside VR were excluded and are part of a separate review looking at virtual patients alone (simulation) [10]. We excluded studies of students and/or practitioners of traditional, alternative, and complementary medicine. We also excluded studies with cross-over design because of the likelihood of a carry-over effect.

We extracted data on the following primary outcomes:

- Learners’ knowledge postintervention: Knowledge is defined as learners’ factual or conceptual understanding measured using change between pre- and posttest scores.
- Learners’ skills postintervention: Skills are defined as learners’ ability to demonstrate a procedure or technique in an educational setting.
- Learners’ attitudes postintervention toward new competences, clinical practice, or patients (eg, recognition of moral and ethical responsibilities toward patients): Attitude is defined as the tendency to respond positively or negatively toward the intervention.
- Learners’ satisfaction postintervention with the learning intervention (eg, retention rates, dropout rates, and survey satisfaction scores): This can be defined as the level of approval when comparing the perceived performance of digital education with one’s expectations.
- Change in learner’s clinical practice or behavior (eg, reduced antibiotic prescriptions and improved clinical diagnosis): This can be defined as any changes in clinical practice after the intervention which results in improvement of the quality of care as well as the clinical outcomes.

We also extracted data on the following secondary outcomes:

- Cost and cost-effectiveness of the intervention
- Patient-related outcomes (eg, patient mortality, patient morbidity, and medication errors)

### Data Sources, Collection, Analysis, and Risk of Bias Assessment

We developed a comprehensive search strategy for MEDLINE (Ovid), Embase (Elsevier), Cochrane Central Register of Controlled Trials (CENTRAL; Wiley), PsycINFO (Ovid), ERIC (Ovid), CINAHL (Ebsco), Web of Science Core Collection, and clinical trial registries (ClinicalTrial.gov and WHO ICTRP). Databases were searched from January 1990 to August 2017. The reason for selecting 1990 as the starting year for our search is that before this year, the use of computers and digital technologies was limited to very basic tasks. There were no language or publication restrictions (see Multimedia Appendix 1).

The search results from different bibliographic databases were combined in a single Endnote library, and duplicate records were removed. Four authors (BMK, NS, JV, and CKN) independently screened the search results and assessed full-text studies for inclusion. Any disagreements were resolved through discussion between the authors. Study authors were contacted for unclear or missing information.

Five reviewers (BMK, NS, JV, CKN, and UD) independently extracted data using a structured data extraction form. Disagreements between review authors were resolved by discussion. We extracted data on the participants, interventions, comparators, and outcomes. If studies had multiple arms, we compared the most interactive intervention arm with the least interactive control arm.

Two reviewers (BMK and NS) independently assessed the risk of bias for randomized controlled trials using the Cochrane *risk of bias* tool [19,21], which included the following domains: random sequence generation, allocation concealment, blinding of outcome assessors, completeness of outcome data, and selective outcome reporting. We also assessed the following additional sources of bias: baseline imbalance and inappropriate administration of an intervention as recommended by the Cochrane Handbook for Systematic Reviews of Interventions [21]. Studies were judged at high risk of bias if there was a high risk of bias for 1 or more key domains and at unclear risk of bias if they had an unclear risk of bias for at least 2 domains.

### Data Synthesis and Analysis

Studies were grouped by outcome and comparison. Comparators included traditional education, other forms of digital education, and other types of VR. We included postintervention outcome data in our review for the sake of consistency as this was the most commonly reported form of findings in the included studies. For continuous outcomes, we summarized the standardized mean differences (SMDs) and associated 95% CIs across studies. We were unable to identify a clinically meaningful interpretation of SMDs specifically for digital education interventions. Therefore, in line with other evidence syntheses of educational research, we interpreted SMDs using the Cohen rule of thumb: <0.2 no effect, 0.2 to 0.5 small effect size, 0.5 to 0.8 medium effect size, and >0.80 a large effect size [22,22]. For dichotomous outcomes, we summarized relative risks and associated 95% CIs across studies. We employed the random-effects model in our meta-analysis. The I^2^ statistic was employed to evaluate heterogeneity, with I^2^<25%, 25% to 75%, and >75% to represent a low, moderate, and high degree of inconsistency, respectively. The meta-analysis was performed using Review Manager 5.3 (Cochrane Library Software, Oxford, UK). Where sufficient data were available, summary SMD and associated 95% CIs were estimated using random-effects meta-analysis [21].

We prepared *Summary of Findings* tables to present a summary of the results and a judgment on the quality of the evidence by using Grading of Recommendations, Assessment, Development and Evaluations methodology. We presented the findings that we were unable to pool, because of lack of data or high heterogeneity, in the form of narrative synthesis.

## Results

### Results of the Search

**Figure 1:**
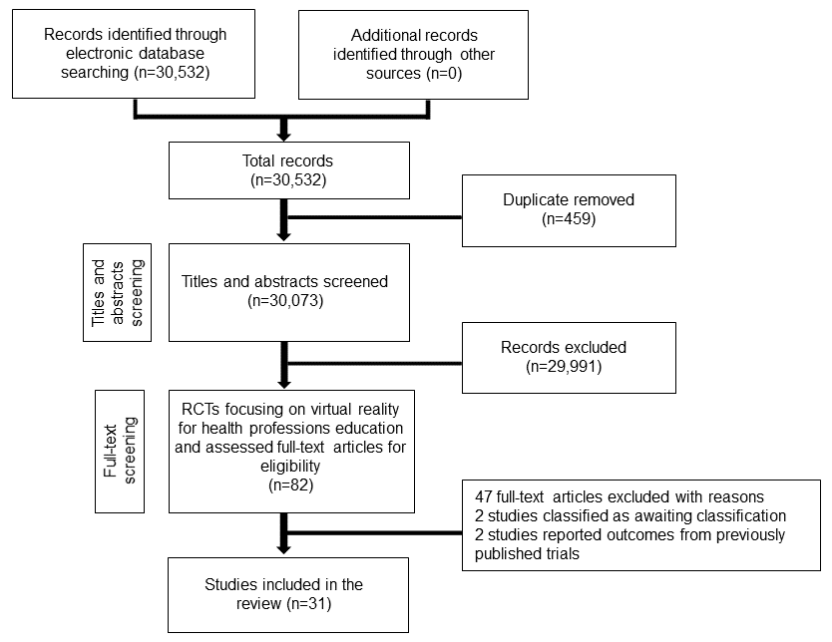
Study flow diagram. RCT: randomized controlled trial.

The searches identified 30,532 unique references; of these, 31 studies (33 reports; 2407 participants) fulfilled the inclusion criteria [11,24-53] (see Figure 1).

### Characteristics of Included Studies

All included studies were conducted in high-income countries. Moreover, 21 studies included only preregistration health professionals. A range of VR educational interventions were evaluated, including 3D models, VP or VHP within virtual worlds, and VR surgical stimulations. Control group interventions ranged from traditional learning (eg, lectures and textbooks) to other digital education interventions (online and offline) and other forms of VR (eg, with limited functions, noninteractivity, or nontutored support; see Multimedia Appendix 2). Although they met the inclusion criteria, some studies did not provide comparable outcome data. Out of the 24 studies assessing knowledge, 1 did not provide any comparable data to estimate the effect of the intervention [44]. Likewise, 2 out of 12 studies assessing skills [29,49], 4 out of 8 studies assessing attitude [38,40,46,50], and 8 out of 12 studies assessing satisfaction [24,29,33,37,48,49,52,53] did not provide comparable data.

### Risk of Bias

**Figure 2:**
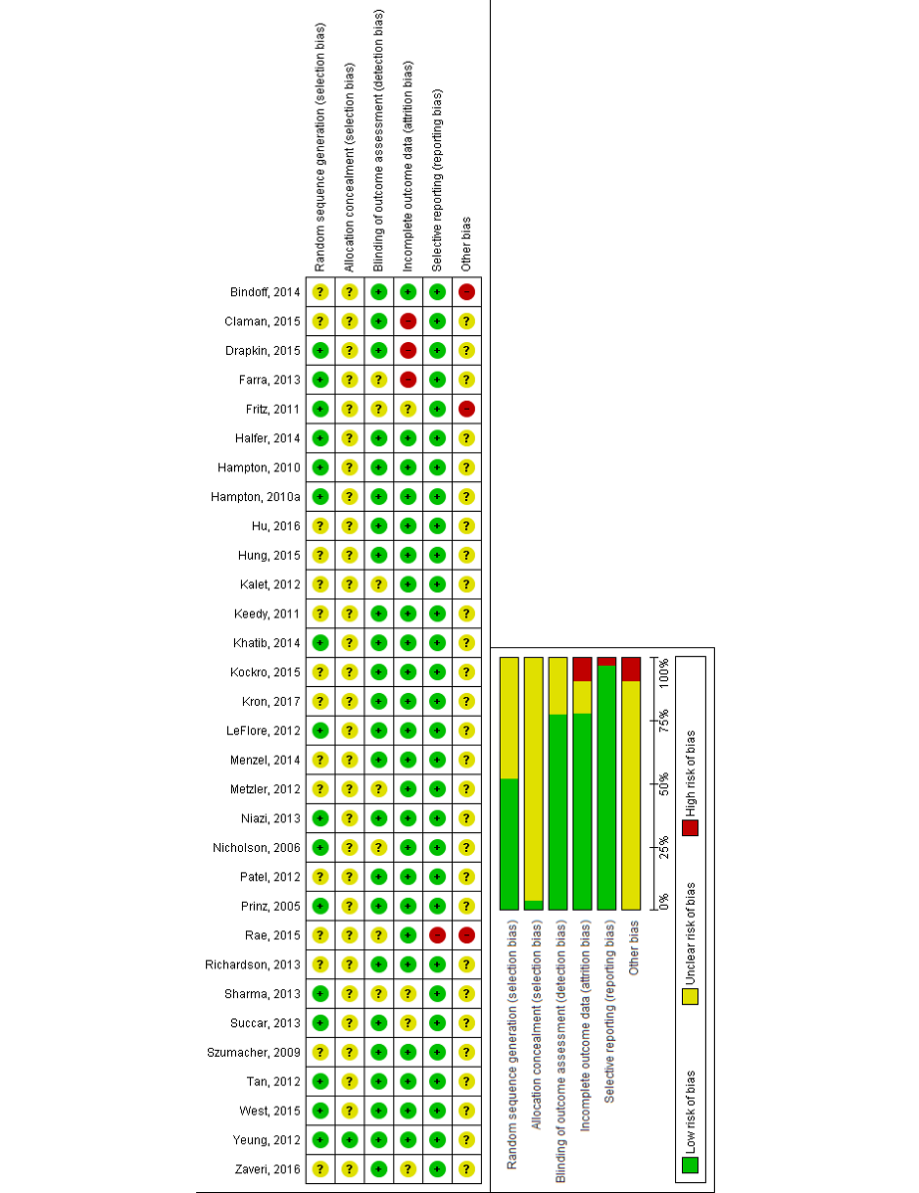
Risk of bias graph and summary.

Overall, studies were judged at unclear or high risk of bias (see Figure 2). Most studies lacked information on randomization, allocation concealment, and participants’ baseline characteristics. Studies were mostly at low risk of bias for blinding of outcome assessment as they provided detailed information on blinding of outcome measures and/or used predetermined assessment tools (multiple choice questions, survey, etc). We judged the studies to be at low risk of detection bias in comparison with traditional education as blinding of participants was impossible because of the use of automated or formalized outcome measurement instruments. However, most of these instruments lacked information on validation. Most studies were judged to be at low risk of attrition and selection bias. Overall, 6 studies were judged at high risk of bias because of reported significant baseline differences in participant characteristics or incomplete outcome data.

### Primary Outcomes

#### Knowledge Outcome

A total of 24 studies (1757 participants) [11,24,26-28,30-32, 34-37,39,41-44,46-48,50-53] assessed knowledge as the primary outcome. Of them, 6 studies focused on postregistration health professionals [30,42,46,47,50,53] and all others focused on preregistration health professionals.

**Figure 3:**
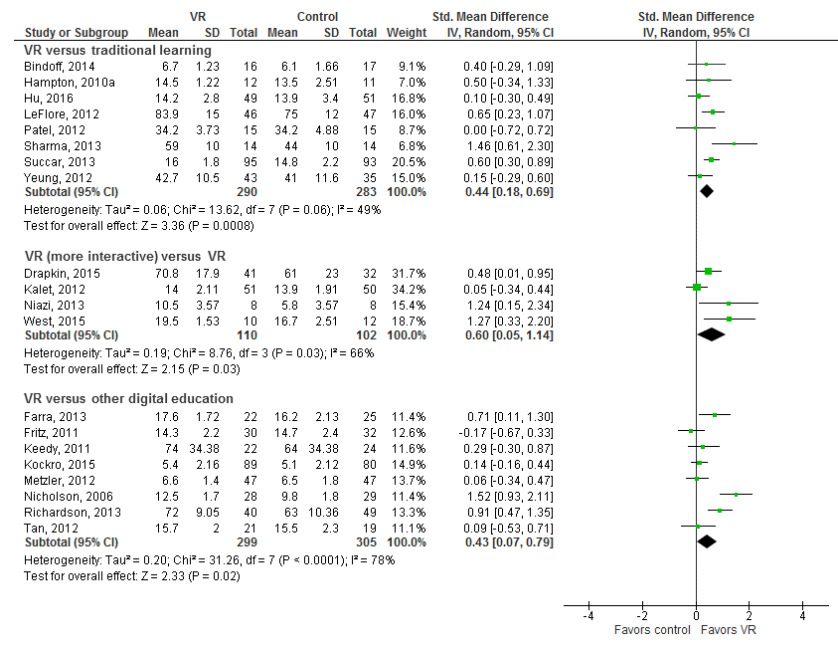
Forest plot for the knowledge outcome (postintervention). df: degrees of freedom; IV: interval variable; random: random effects model; VR: virtual reality.

The effectiveness of VR interventions was compared with traditional learning (via two-dimensional [2D] images, textbooks, and lectures) in 9 studies (659 participants) [24,30,32,36,39,43,47,48,52] (Table 1). Overall, studies suggested a slight improvement in knowledge with VR compared with traditional learning (SMD=0.44; 95% CI 0.18-0.69; I^2^=49%; 603 participants [8 studies]; moderate certainty evidence; see Figure 3).

A total of 10 studies (812 participants) compared VR with other forms of digital education (comprising 2D images on a screen, simple videos, or Web-based teaching) [11,27,28,35,37,41, 44,46,50,53] (see Table 2). The overall pooled estimate of 8 studies that compared different types of VR (such as computer 3D model and virtual world) with different controls (ie, computer-based 2D learning or online module or video-based learning) reported higher postintervention knowledge scores in the intervention groups over the control groups (SMD=0.43; 95% CI 0.07-0.79; I^2^=78%; 608 participants; low certainty evidence; see Figure 3). Additionally, 4 studies compared 3D models with different levels of interactivity (243 participants) [26,34,42,51]. Models with higher interactivity were associated with greater improvements in knowledge than those with less interactivity. The overall pooled estimate of the 4 studies reported higher postintervention knowledge score in the intervention groups with higher interactivity compared with the less interactive controls (SMD=0.60; 95% CI 0.05-1.14; I^2^=66%; moderate effect size; low certainty evidence; see Figure 3). A total of 3 studies could not be included in the meta-analysis: 1 study lacked data [44], whereas the other 2 studies reported a mean change score, favoring the VR group [36] or other digital education intervention [53].

**Table 1:** Summary of findings table: virtual reality compared with traditional learning.

Outcomes^a^	Illustrative comparative risks (95% CI)	Participants (n)	Studies (n)	Quality of evidence (GRADE^b^)	Comments
Postintervention knowledge scores: measured via MCQs^c^ or quiz. Follow-up: immediate postintervention only	The mean knowledge score in the intervention group was 0.44 SDs higher (0.18 to 0.69 higher) than the mean score in the traditional learning group	603	8	Moderate^d^	1 study [36] reported mean change scores within the group, and hence, the study data were excluded from the pooled analysis
Postintervention skill scores: measured via survey and OSCE^e^. Follow-up duration: immediate postintervention only	The mean skill score in the intervention group was 1.12 SDs higher (0.81 to 1.43 higher) than the mean score in the traditional learning group	235	4	Moderate^d^	3 studies were excluded from the analysis as 1 study reported incomplete outcome data [29], 1 study assessed mixed outcomes [36], and 1 study reported self-reported outcome data [24]
Postintervention attitude scores: measured via survey. Follow-up duration: immediate postintervention only	The mean attitudinal score in the intervention group was 0.19 SDs higher (−0.35 lower to 0.73 higher) than the mean score in the traditional learning group	83	2	Moderate^d^	N/A^f^
Postintervention satisfaction scores: measured via survey. Follow-up duration: immediate postintervention only	Not estimable	100	1	Low^d,g^	5 studies [24,29,33,48,52] reported incomplete outcome data or lacked comparable data. Therefore, these studies were excluded from the analysis.

^a^Patient or population: health professionals; settings: universities and hospitals; intervention: virtual reality; comparison: traditional learning (face-to-face lecture, textbooks, etc).

^b^GRADE (Grading of Recommendations, Assessment, Development and Evaluations) Working Group grades of evidence. High quality: further research is very unlikely to change our confidence in the estimate of effect; moderate quality: further research is likely to have an important impact on our confidence in the estimate of effect and may change the estimate; low quality: further research is very likely to have an important impact on our confidence in the estimate of effect and is likely to change the estimate; and very low quality: we are very uncertain about the estimate.

^c^MCQs: multiple choice questions.

^d^Downgraded by 1 level for study limitations: the risk of bias was unclear or high in most included studies (−1).

^e^OSCE: objective structured clinical examination.

^f^N/A: not applicable.

^g^Downgraded as results were obtained from a single small study (−1).

#### Skill Outcome

**Figure 4:**
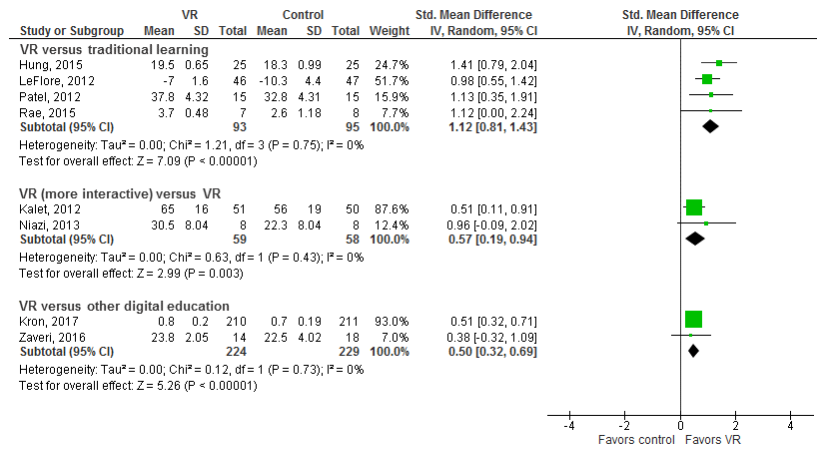
Forest plot for the skills outcome (postintervention). df: degrees of freedom; IV: interval variable; random: random effects model; VR: virtual reality.

A total of 12 studies (1011 participants) assessed skills as an outcome [24,29,33,34,36,38,39,42,43,45,49,53]. Of which, 7 studies compared VR-based interventions with traditional learning (comprising paper- or textbook-based education and didactic lectures; 354 participants) [24,29,33,36,39,43,45], and the overall pooled estimate of 4 studies showed a large improvement in postintervention cognitive skill scores in the intervention groups compared with the controls (SMD=1.12; 95% CI 0.81-1.43; I^2^=0%, 235 participants; large effect size; moderate certainty evidence; see Figure 4 and Table 1). Additionally, 3 studies compared the effectiveness of different types of VR on cognitive skills acquisition (190 participants) [34,42,49]. We were able to pool the findings from 2 studies favoring more interactive VR (SMD=0.57; 95% CI 0.19-0.94; I^2^=0%; moderate effect size; low certainty evidence). Two studies compared VR with other forms of digital education on skills, favoring the VR group (SMD=0.5; 95% CI 0.32-0.69; I^2^=0%; 467 participants; moderate effect size; low certainty evidence; see Table 2). A total of 4 studies could not be included in the meta-analysis: 2 studies reported incomplete outcome data [29,49], 1 study assessed mixed outcomes [36], and 1 study reported self-reported outcome data [24].

#### Attitude Outcome

A total of 8 studies (762 participants) [25,30,31,38,40,43,46,50] assessed attitude as an outcome. Of them, 2 studies comparing VR-based interventions with traditional learning (small group teaching and didactic lectures; 83 participants) [30,43] reported no difference between the groups on postintervention attitude scores (SMD=0.19; 95% CI−0.35 to 0.73; I^2^=0%; moderate certainty evidence; see Table 1). One study compared blended learning with traditional learning (43 participants) [30] and reported higher postinterventional attitude score (large effect size) toward the intervention (SMD=1.11; 95% CI 0.46-1.75). Additionally, 5 studies (636 participants) [25,38,40,46,50] that compared VR with other digital education interventions reported that most of the studies had incomplete outcome data.

**Table 2:** Summary of findings table: virtual reality compared with other digital education interventions.

Outcomes^a^	Illustrative comparative risks (95% CI)	Participants (n)	Studies (n)	Quality of evidence (GRADE^b^)	Comments
Postintervention knowledge score: measured via MCQs^c^ and questionnaires. Follow-up duration: immediate postintervention to 6 months	The mean knowledge score in the intervention group was 0.43 SDs higher (0.07 to 0.79 higher) than the mean score in the other digital education interventions	608	8	Low^d,e^	1 study (32 participants) presented mean change score and favored VR group compared with the control group [53], and 1 study (172 participants) compared VR with computer-based video (2D) and presented incomplete outcome data [44]
Postintervention skills score: measured via scenario-based skills assessment. Follow-up duration: immediate postintervention only	The mean skill score in the intervention group was 0.5 SDs higher (0.32 to 0.69 higher) than the mean score in the other digital education interventions	467	2	Moderate^d^	N/A^f^
Postintervention attitude: measured via survey and questionnaire. Follow-up duration: immediate postintervention only.	Not estimable	21	1	Low^d,g^	4 studies [38,40,46,50] reported incomplete outcome data or lacked comparable data. Therefore, these studies were excluded from the analysis.
Postintervention satisfaction: measured via MCQs, survey, and questionnaire. Duration: immediate postintervention only	The mean satisfaction score in the intervention group was 0.2 SDs higher (−0.71 lower to 1.11 higher) than the mean score in the other digital education interventions	218	2	Low^d,e^	2 studies [37,53] reported incomplete outcome data or lacked comparable data. Therefore, these studies were excluded from the analysis.

^a^Patient or population: Health professionals; Settings: Universities and hospitals; Intervention: Virtual reality; Comparison: Other digital education interventions (such as online learning, computer-based video, etc).

^b^GRADE (Grading of Recommendations, Assessment, Development and Evaluations) Working Group grades of evidence. High quality: Further research is very unlikely to change our confidence in the estimate of effect; Moderate quality: Further research is likely to have an important impact on our confidence in the estimate of effect and may change the estimate; Low quality: Further research is very likely to have an important impact on our confidence in the estimate of effect and is likely to change the estimate; and Very low quality: We are very uncertain about the estimate.

^c^MCQs: multiple choice questions.

^d^Downgraded by 1 level for study limitations (−1): the risk of bias was unclear or high in most included studies.

^e^Downgraded by 1 level for inconsistency (−1): the heterogeneity between studies is high with large variations in effect and lack of overlap among confidence intervals.

^f^N/A: not applicable.

^g^Downgraded as results were obtained from a single small study (−1).

#### Satisfaction Outcome

A total of 12 studies (991 participants) [24,26,29,32,33,35, 37,44,48,49,52,53] assessed satisfaction, mostly only for the intervention group. Only 4 studies compared satisfaction in the intervention and control groups and largely reported no difference between the study groups.

### Secondary Outcomes

Halfer et al (30 participants) [29] assessed the use of VR versus traditional paper floor plans of the hospital to prepare nurses for wayfinding in a new hospital building. A cost analysis showed that a virtual hospital-based approach increased development costs but provided increased value during implementation by reducing staff time needed for practicing wayfinding skills. The paper describes that the real-world paper floor plan approach had a development cost of US $40,000 and the implementation cost was US $530,000, bringing the total cost to US $570,000. In comparison, the virtual world would cost US $220,000 for development and US $201,000 for implementation, bringing the total to US $421,000.

Zaveri et al (32 participants) [53] assessed the effectiveness of a VR module (Second Life, Linden Lab) in teaching preparation and management of sedation procedures, compared with online learning. Development of the module occurred over 2 years of interactions with a software consultant and utilized a US $40,000 grant to create VR scenarios. Cost of the control group (online training) was not presented, and hence, no formal comparison was made.

No information on patient-related outcomes, behavior change, and unintended or adverse effects of VR on the patient or the learner were reported in any of the studies.

## Discussion

### Principal Findings

This systematic review assessed the effectiveness of VR interventions for health professions education. We found evidence showing a small improvement in knowledge and moderate-to-large improvement in skills in learners taking part in VR interventions compared with traditional or other forms of digital learning. Compared with less interactive interventions, more interactive VR interventions seem to moderately improve participants’ knowledge and skills. The findings for attitude and satisfaction outcomes are inconclusive because of incomplete outcome data. None of the included studies reported any patient-related outcomes, behavior change, as well as unintended or adverse effects of the VR on the patients or the learners. Only 2 studies assessed the cost of setup and maintenance of the VR as a secondary outcome without making any formal comparisons.

Overall, the risk of bias for most studies was judged to be unclear (because of a lack of information), with some instances of potentially high risk of attrition, reporting, and other bias identified. The quality of the evidence ranges from low to moderate for knowledge, skills, attitude, and satisfaction outcomes because of the unclear and high risks of bias and inconsistency, that is, heterogeneity in study results as well as in types of participants, interventions, and outcome measurement instruments [54].

The fact that no included studies were published before 2005 suggests that VR is an emerging educational strategy, attracting increasing levels of interest. The included studies were mainly conducted among doctors, nurses, and students pursuing their medical degree. Limited studies on pharmacists, dentists, and other allied health professionals suggest more research is needed on the use of VR among these groups of health professionals. Additionally, the majority of interventions studied were not part of a regular curriculum and none of the studies mentioned the use of learning theories to design the VR-based intervention or develop clinical competencies. This is an important aspect of designing any curriculum, and hence, applicability of the included studies might only be limited to their current setting and may not be generalizable to other geographic or socioeconomic backgrounds. Furthermore, most studies evaluated participants’ knowledge, and skills assessed may not translate directly into clinical competencies.

Although the included studies encompassed a range of participants and interventions, a lack of consistent methodological approach and studies conducted in any one health care discipline makes it difficult to draw any meaningful conclusions. There is also a distinct lack of data from low- or middle-income countries, which reduces applicability to those contexts that are most in need of innovative educational strategies. In addition, only 2 studies assessed the cost of setup and maintenance of the VR-based intervention, whereas none of the included studies assessed cost-effectiveness. Thus, no conclusions regarding costing and cost-effectiveness can be made at this point either. There was also a lack of information on patient-related outcomes, behavior change, and unintended or adverse effects of VR on the patients as well as the learner, which needs to be addressed.

Majority of the included studies assessed the effectiveness of nonimmersive VR, and there is a need to explore more on the effects of VR with different level of immersion as well as interactivity on the outcomes of interest. In our review, most of the studies assessing attitude and satisfaction outcomes reported incomplete outcome or incomparable outcome data, and there is a need for primary studies focusing on these outcomes. Finally, there is a need to standardize the methods for reporting meaningful and the most accurate data on the outcomes as most of the included studies reported postintervention mean scores rather than change scores on the outcomes, which limits the accuracy of the findings for the reported outcomes.

### Strengths and Limitations

Our review provides the most up-to-date evidence on the effectiveness of different types of VR in health professions education. We conducted a comprehensive search across different databases including gray literature sources and followed the Cochrane gold standard methodology while conducting this systematic review. Our review also has several limitations. The included studies largely reported postintervention data, so we could not calculate pre- to postintervention change or ascertain whether the intervention groups were matched at baseline for key characteristics and outcome measure scores. We were also unable to perform the prespecified subgroup analysis because of limited data from the primary studies.

### Conclusions

As an emerging and versatile technology, VR has the potential to transform health professions education. Our findings show that when compared with traditional education or other types of digital education, such as online or offline digital education, VR may improve postintervention knowledge and skills. VR with higher interactivity showed more effectiveness compared with less interactive VR for postintervention knowledge and skill outcomes. Further research should evaluate the effectiveness of more immersive and interactive forms of VR in a variety of settings and evaluate outcomes such as attitude, satisfaction, untoward effects of VR, cost-effectiveness, and change in clinical practice or behavior.

## Appendix

Multimedia Appendix 1 MEDLINE (Ovid) search strategy.

Multimedia Appendix 2 Characteristics of included studies.

## Abbreviations

2D: two-dimensional
3D: three-dimensional
GRADE: Grading of Recommendations, Assessment, Development and Evaluations
MCQ: multiple choice question
OSCE: objective structured clinical examination
SMD: standardized mean difference
VHP: virtual health professional
VP: virtual patient
VR: virtual reality
WHO: World Health Organization
