# Supplementary material for: Virtual Reality for Health Professions Education: Systematic Review and Meta-Analysis by the Digital Health Education Collaboration
Source: J Med Internet Res. 2019 Jan 22;21(1):e12959. doi: 10.2196/12959 (PMC6362387; doi:10.2196/12959)
Supplement: Multimedia Appendix 2 [file jmir_v21i1e12959_app2.pdf]

Multimedia Appendix 2: Characteristics of included studies.

Authors, design, country	Number of participants	Type of comparison	VR[a] type	Setting, funding	Type of participants (pre- or postregistration)	Learning objectives
Bindoff 2014 [24], RCT[b], Australia	33	VR versus traditional (paper-based approach)	VP/VHP[c] within VR	University, nonindustrial funding	Third- and fourth-year pharmacy students (preregistration)	To improve pharmacy students’ knowledge, skills, and attitude regarding community pharmacy practice
Claman 2015 [25], RCT, USA	21	VR versus online/computer-based learning (blackboard online platform)	VP/VHP within VR	University, nonindustrial funding	Family nurse practitioner students (preregistration)	To improve nursing students’ attitude toward the VP/VHP within VR
Drapkin 2015 [26], RCT, USA	62	VR versus VR (3D[d] models)	3D models	University, N/A[e]	First-year medical students (preregistration)	To improve medical students’ knowledge to identify subcortical structures on magnetic resonance images as well as to assess medical students’ satisfaction with the VR
Farra 2013 [27], RCT, USA	47	VR versus other digital education (Web-based teaching method)	VP/VHP within VR	University, industry, and public sponsorship	Nursing students in their final year of study (preregistration)	To improve nursing students’ knowledge regarding disaster training
Fritz 2011 [28], RCT, Canada	62	VR versus other digital education (2D images)	3D models	University, nonindustrial funding	Medicine students from year 1 to 4, dentistry and physical therapy students (preregistration)	To improve students’ knowledge on laryngeal anatomy at short-term as well as to assess long-term knowledge retention
Halfer 2014 [29], RCT, USA	30	VR versus traditional (paper-based approach)	Virtual world	Hospital, N/A	Registered nurses (postregistration)	To improve nurses’ navigation skills within a hospital (wayfinding through a hospital)
Hampton 2010 [30],	43	VR (blended)	3D models	University, industry, and	Third- and fourth-year medical students	To improve medical students’ knowledge on

RCT, USA		versus traditional (learning via lectures)		public sponsorship	(preregistration)	female pelvic anatomy as well as to assess attitude toward VR intervention
Hampton 2010a [31], RCT, USA	23	VR versus traditional (learning via lectures)	3D models	University, industry, and public sponsorship	Obstetrics/gynecology residents (postregistration)	To improve residents' knowledge on female pelvic anatomy and to assess attitude toward VR intervention
Hu 2016 [32], RCT, USA	100	VR versus traditional (written text)	3D models	University, no funding received	Medical students (preregistration)	To improve medical students' knowledge on laryngeal anatomy as well as to assess medical residents' satisfaction with the intervention outcome
Hung 2015 [33], RCT, Hong Kong	50	VR versus traditional (usual training)	VP/VHP within VR	University, N/A	Registered nurses, enrolled nurses, and health care assistants at OPD[f] (postregistration)	To improve participants' skills regarding the use of personal protective equipment as well as to assess participants' satisfaction with the intervention
Kalet 2012 [34], RCT, USA	143	VR versus VR (3D models)	3D models	University, nonindustrial funding	Second-year medical students (preregistration)	To improve medical students' knowledge and skill on abdominal examination
Keedy 2011 [35], RCT, USA	46	VR versus other digital education (computer-based 2D images)	3D models	University, N/A	First- and fourth-year medical students (preregistration)	To improve medical students' knowledge on liver anatomy as well as to assess medical students' satisfaction outcome with VR intervention
Khatib 2014 [36], RCT, UK	56	VR versus traditional (textbooks)	VR surgical simulation	University, nonindustrial funding	Medical students in their clinical years (preregistration)	To improve medical students' knowledge and skills with inguinal hernia repair simulation
Kockro 2015 [37], RCT, Germany	169	VR versus other digital education (2D[g]	3D models	University, nonindustrial funding	Second-year medical students (preregistration)	To improve medical students' knowledge on neuroanatomy and to assess satisfaction with

		images)				VR intervention
Kron 2017 [38], RCT, USA	435	VR versus other digital education (computer-based learning)	3D models	University, industrial funding	Second-year medical students (preregistration)	To improve communication skills of the medical students as well as to assess attitude toward the intervention outcome
LeFlore 2012 [39], RCT, USA	93	VR versus traditional (learning via lectures)	VP/VHP within VR	Hospital, N/A	Undergraduate registered nursing students (preregistration)	To improve nursing students' knowledge and skills regarding pediatric respiratory diseases
Menzel 2014 [40], RCT, USA	51	VR versus other digital education (Web-based learning)	VP/VHP within VR	University, nonindustrial funding	Nursing students (preregistration)	To improve nursing students' attitude toward poverty (simulation in VR environment)
Metzler 2012 [41], RCT, Germany	94	VR versus other digital education (2D images)	3D models	University, N/A	Fourth- and fifth-year medical students (preregistration)	To improve medical students' knowledge on liver anatomy
Niazi 2013 [42], RCT, Canada	16	VR versus VR (3D models)	3D models	University, industrial funding	Anesthesia residents (postregistration)	To improve residents' knowledge on lumbar anatomy and to improve skills regarding lumbar puncture
Nicholson 2006 [11], RCT, USA	61	VR versus other digital education (Web-based tutorial)	3D models	University, nonindustrial funding	First-year medical students (preregistration)	To improve medical students' knowledge on ear anatomy
Patel 2012 [43], RCT, UK	60	VR versus traditional (no further training)	VP/VHP within VR	University, nonindustrial funding	First-year medical students (preregistration)	To improve medical students' knowledge and skills outcomes regarding performance in the operating theatre as well as to assess medical students' attitude toward operating theatre
Prinz 2005 [44], RCT, UK	172	VR versus other digital education	3D models	University, N/A	Medical students (preregistration)	To improve medical students' knowledge and attitude on

		(computer-based video)				ophthalmology
Rae 2015 [45], RCT, UK	32	VR versus traditional (learning via textbooks)	VR surgical simulation	University, nonindustrial funding	Foundation doctor trainees and core surgical trainees (postregistration)	To improve medical doctors' knowledge and skills with inguinal hernia repair simulation
Richardson 2013 [46], RCT, USA	89	VR versus other digital education (2D images)	3D models	University, nonindustrial funding	Second-year students enrolled in a 4-year masters of pharmacy program (postregistration)	To improve pharmacy students' knowledge on pharmacology and attitude toward the VR intervention
Sharma 2013 [47], RCT, UK	28	VR versus traditional (learning via textbooks)	3D models	Hospital, no funding received	Anesthetic trainees (specialty training year 4 to year 6; postregistration)	To improve anesthetic trainees' knowledge on transoesophageal echocardiography
Succar 2013 [48], RCT, Australia	188	VR versus traditional (usual learning)	VP/VHP within VR	University, nonindustrial funding	Medical students (preregistration)	To improve medical students' knowledge and satisfaction outcome on ophthalmology training in VR
Szumacher 2009 [49], RCT, Canada	31	VR versus VR (3D models)	3D models	University, N/A	Radiation oncology residents and fellows as well as third-year radiotherapy students and radiation therapists (both pre- and postregistration)	To improve participants' skills and satisfaction with radiology training (prostate and rectal contouring)
Tan 2012 [50], RCT, Canada	40	VR versus other digital education (2D images)	3D models	University, N/A	First- and second-year surgical and anesthesia medical residents (postregistration)	To improve participants' knowledge and attitude outcomes in laryngeal anatomy
West 2015 [51], RCT, Denmark	22	VR versus VR (nontutored computer simulation program)	3D models	University, N/A	Medical students (preregistration)	To improve medical students' knowledge in temporal bone anatomy
Yeung 2012 [52], RCT, UK	78	VR versus traditional (learning via textbook)	3D models	University, N/A	Second-year students from health science course in anatomy and physiology (preregistration)	To improve participants' knowledge in cranial nerve anatomy as well as to improve participants' satisfaction with VR intervention
Zaveri 2016	32	VR versus	VP/VHP	Hospital,	Pediatrics residents	To improve pediatrics

[53], RCT, USA		other digital education (online sources)	within VR	nonindustrial funding	(postregistration)	residents' knowledge, skill, and satisfaction outcome with pediatric sedation

[a]VR: virtual reality.

[b]RCT: randomized controlled trials.

[c]VP/VHP: virtual patient/virtual health professionals.

[d]3D: three-dimensional.

[e]N/A: not available.

[f]OPD: out patient department.

[g]2D: two-dimensional.
